# Rapid Antigen Group A Streptococcus Test to Diagnose Pharyngitis: A Systematic Review and Meta-Analysis

**DOI:** 10.1371/journal.pone.0111727

**Published:** 2014-11-04

**Authors:** Emily H. Stewart, Brian Davis, B. Lee Clemans-Taylor, Benjamin Littenberg, Carlos A. Estrada, Robert M. Centor

**Affiliations:** 1 Walter Reed National Military Medical Center, Bethesda, Maryland, United States of America; 2 University of Texas Southwestern Medical Center, Dallas, Texas, United States of America; 3 The University of Alabama at Birmingham, Huntsville Campus, Huntsville, Alabama, United States of America; 4 University of Vermont, Burlington, Vermont, United States of America; 5 University of Alabama at Birmingham, Birmingham, Alabama, United States of America; 6 Birmingham Veterans Affairs Medical Center and Veterans Affairs Quality Scholar Program, Birmingham, Alabama, United States of America; Wake Forest University School of Medicine, United States of America

## Abstract

**Background:**

Pharyngitis management guidelines include estimates of the test characteristics of rapid antigen streptococcus tests (RAST) using a non-systematic approach.

**Objective:**

To examine the sensitivity and specificity, and sources of variability, of RAST for diagnosing group A streptococcal (GAS) pharyngitis.

**Data Sources:**

MEDLINE, Cochrane Reviews, Centre for Reviews and Dissemination, Scopus, SciELO, CINAHL, guidelines, 2000–2012.

**Study Selection:**

Culture as reference standard, all languages.

**Data Extraction and Synthesis:**

Study characteristics, quality.

**Main Outcome(s) and Measure(s):**

Sensitivity, specificity.

**Results:**

We included 59 studies encompassing 55,766 patients. Forty three studies (18,464 patients) fulfilled the higher quality definition (at least 50 patients, prospective data collection, and no significant biases) and 16 (35,634 patients) did not. For the higher quality immunochromatographic methods in children (10,325 patients), heterogeneity was high for sensitivity (inconsistency [*I^2^*] 88%) and specificity (*I^2^* 86%). For enzyme immunoassay in children (342 patients), the pooled sensitivity was 86% (95% CI, 79–92%) and the pooled specificity was 92% (95% CI, 88–95%). For the higher quality immunochromatographic methods in the adult population (1,216 patients), the pooled sensitivity was 91% (95% CI, 87 to 94%) and the pooled specificity was 93% (95% CI, 92 to 95%); however, heterogeneity was modest for sensitivity (*I^2^* 61%) and specificity (*I^2^* 72%). For enzyme immunoassay in the adult population (333 patients), the pooled sensitivity was 86% (95% CI, 81–91%) and the pooled specificity was 97% (95% CI, 96 to 99%); however, heterogeneity was high for sensitivity and specificity (both, *I^2^* 88%).

**Conclusions:**

RAST immunochromatographic methods appear to be very sensitive and highly specific to diagnose group A streptococcal pharyngitis among adults but not in children. We could not identify sources of variability among higher quality studies. The present systematic review provides the best evidence for the wide range of sensitivity included in current guidelines.

## Introduction

Rapid antigen testing to detect group A Streptococcal (GAS) infection provides important information for the antibiotic decision making for patients presenting with acute pharyngitis. Pharyngitis accounts for over 13 million office visits annually in the United States [1], highlighting the importance of these decisions. Patients with GAS pharyngitis can develop either suppurative or non-suppurative complications. Given the importance of chronic rheumatic fever in the 1950s, preventing acute rheumatic fever became the main focus of treating pharyngitis at the time. While the incidence of acute rheumatic fever has decreased, the focus in patients with acute pharyngitis is on treating GAS infections to decrease suppurative complications (especially peritonsillar abscess), decrease person-to-person spread, and to shorten symptom duration.

The Infectious Diseases Society of America (IDSA) guideline [2] on streptococcal pharyngitis recommends using a rapid test in patients with a modest probability of GAS infection, treating those with a positive rapid test and withholding antibiotics in rapid test negative patients. The guideline recommends culturing rapid test negative children and treating patients having positive cultures; the guideline does not recommend culturing the rapid test negative adults given the lower prevalence and significantly reduced chance of non-suppurative complications of the disease in the adult population [2], unless the clinician wishes to increase diagnostic sensitivity.

The IDSA guidelines and reviews have documented excellent specificity of rapid antigen streptococcal testing; however, the sensitivity estimate varies from 70% to 95% [3]–[6]. These reports did not apply a systematic approach to make these estimates. A systematic review published in Spanish [7] did not examine potential sources of heterogeneity.

The present study explores the variability of sensitivity and specificity using a systematic approach; the goal of this systematic review was to identify accurate, unbiased estimates of rapid antigen streptococcus test (RAST) characteristics for children, adults, and RAST variety.

## Materials and Methods

We performed a systemic review and meta-analysis of the performance of various rapid antigen streptococcus tests to diagnose Group A streptococcal pharyngitis in adult and pediatric populations using standard guidelines for diagnostic studies [8]. We also used the Preferred Reporting Items for Systematic reviews and Meta-Analyses for reporting [9]. We limited our study to more recent publications because the technology of rapid antigen testing has improved over the years and to exclude tests no longer used.

### Data Sources and Searches

In preparation for identifying search terms, a professional medical librarian (BLCT) searched the National Library of Medicine’s MEDLINE electronic database from January 2000 to April 2012 using PubMed, limiting the search to the English language only and meta-analyses or systematic reviews. We then ran preliminary test searches to identify all possible terms necessary to design a comprehensive and systematic search strategy. Finally, we used medical subject headings (MeSH terms) and text words to search for three main concept areas: target condition, index test, and test characteristics (see Methods S1). The three main concepts were combined using AND as the Boolean operator. In addition, we supplemented the search with the PubMed/MEDLINE’s Clinical Queries feature to combine the target condition with the diagnosis/broad automatic filter. We completed the first search on April 11, 2012 and repeated the same search strategy on October 26, 2012 to update the search and expand the scope by including non-English citations.

We also checked online through PubMed/MEDLINE and hand searched several major infectious disease, clinical microbiology, and pediatric textbooks for updates to current guidelines on the use of rapid antigen detection tests in the diagnosis of group a beta-hemolytic streptococcus including the Infectious Diseases Society of America (IDSA), American Heart Association, American Academy of Pediatrics, the American College of Physicians (ACP), the Centers for Disease Control, and the American Academy of Family Physicians. In addition, we searched the electronic sources Cochrane Reviews, Centre for Reviews and Dissemination [10], UpToDate, DynaMed, and Essential Evidence Plus; we also reviewed references from personal files (RMC, one of the authors). We also reviewed references from cost-effectiveness studies.

We did not include data from package inserts of commercially available RAST as study characteristics were not included [11]. Finally, we searched the electronic sources Scopus [12], SciELO (Scientific Electronic Library Online) [13], and CINAHL (Cumulative Index to Nursing and Allied Health Literature) on December 6, 2012 for studies published after 2000 without language limits.

### Study Selection

Two of the authors independently reviewed the titles of the initial search results and excluded titles that were not relevant, non-English, lacking a RAST, review articles, studies that lacked culture as reference standard, or other reasons (ex: duplicate publications, non-human studies, case reports, letters to the editor, no data reported). Discrepancies were included in the second review. A third author reviewed all excluded titles (CAE). In the second review, two authors independently read the titles and abstracts for the same exclusion criteria; a third author resolved conflicts. In the third review, one author read the articles and another confirmed the excluded articles (a third author resolved conflicts during this step). We excluded articles that did not use a culture reference standard.

### Data Extraction and Quality Assessment

We recorded country of study, funding source, index test location (point-of-care or laboratory), number of swabs for the reference test (one or two), culture medium, age of population, setting (outpatient clinic, student health, emergency room), inclusion and exclusion criteria, and study design (prospective, retrospective). We constructed 2×2 contingency tables (true positives, false positives, false negatives, true negatives) from the published data for the main study results and for any subgroups reported. We excluded articles where a 2×2 contingency table could not be calculated from the published data. We used the Quality Assessment of Diagnostic Accuracy Studies (QUADAS) checklist to assess methodological quality of the studies [14]. Each of two authors abstracted data for half of the studies selected; at the end, the other author reviewed the abstracted data for independent verification.

### Data Synthesis and Analysis

Based on the 2×2 contingency table, we computed prevalence, sensitivity, and specificity for each study and each subgroup.

We examined heterogeneity with graphical methods using coupled forest plots of sensitivity and specificity and hierarchical summary receiver-operating characteristic (HSROC) curves [8], [15]–[17]. The HSROC uses a random-effects model and accounts for the relationship between sensitivity and specificity in each study. The HSROC analyses provide estimates of uncertainty that includes a 95% confidence region (for the summary estimate) and a 95% prediction region (for a forecast of the sensitivity and specificity in a future study) [18]. Wider prediction regions suggest significant heterogeneity [8], [17]. The summary ROC may also identify a threshold effect, suggested by a shoulder-like appearance of the curve, that could explain heterogeneity between studies [8].

We also used the inconsistency (*I^2^*) value to examine heterogeneity and regarded values as low, moderate, or high heterogeneity for values of 25%, 50%, or 75% (respectively). However, a recent review noted limitations of the *I^2^* as it does not account for the correlation between sensitivity and specificity, does not account for variation explained by threshold effects, and overestimates heterogeneity [17]. We include pooled estimates of sensitivity and specificity in the results section when values were deemed homogeneous enough or for illustration purposes.

We explored heterogeneity, a-priori, by examining studies of highest quality, defined as those with at least 50 patients, prospective data collection, and three items of the QUADAS methodological quality criteria [14]: “Did the whole sample or a random selection of the sample, receive verification using a reference standard for diagnosis?” (partial verification avoided), “Did patients receive the same reference standard regardless of the index test results?” (differential verification avoided), and “Was the reference standard independent of the index test (i.e.: the index test did not form part of the reference standard)?” (incorporation bias avoided). We did not require blinding of the reference standard or the index test to define a study as high quality.

We examined publication bias with the Deeks’ funnel plots and tested asymmetry with linear regression of log diagnostic odds ratios (DOR) on the inverse root of the effective sample size [19]. In the absence of publication bias, studies of smaller sample size would have a wider distribution of results (in diagnostic test studies, DOR) due to random variation as compared to studies with larger sample size that would have a narrower distribution of results. A non-vertical line with a p value<0.10 for the slope of the coefficient indicates asymmetry and suggests publication bias. The Deeks’ funnel plot method [19] overcomes limitations of other methodologies.

We also explored heterogeneity post-hoc. The purpose of these analyses was to identify study sub-groups with sufficient clinical and statistical homogeneity to calculate summary estimates of sensitivity and specificity. We limited the exploratory analyses to the highest quality studies as defined above. We analyzed age groups separately, exclusive pediatric population vs. other, as the clinical features and epidemiology are different. We also analyzed separately by index test methodology (immuno-chromatographic, enzyme immunoassay, optical immune-assay). Finally, we explored sponsorship (commercial vs. none or none reported), location of performance of the index test (laboratory vs. point-of-care or not reported), risk score (Centor or McIassac), location of care (outpatient vs. emergency room), publication year (2000–2005 vs. 2006–2012), prevalence (by tertiles), and region (USA/Canada vs. Europe vs. other). We also performed meta-regression to estimate the independent contribution of the variables listed above that may explain heterogeneity [20].

We used STATA 11.2 software (College Station, Texas, USA) and the midas [20] and metandi [18] modules for statistical analyses.

## Results

### Study selection

**Figure 1:**
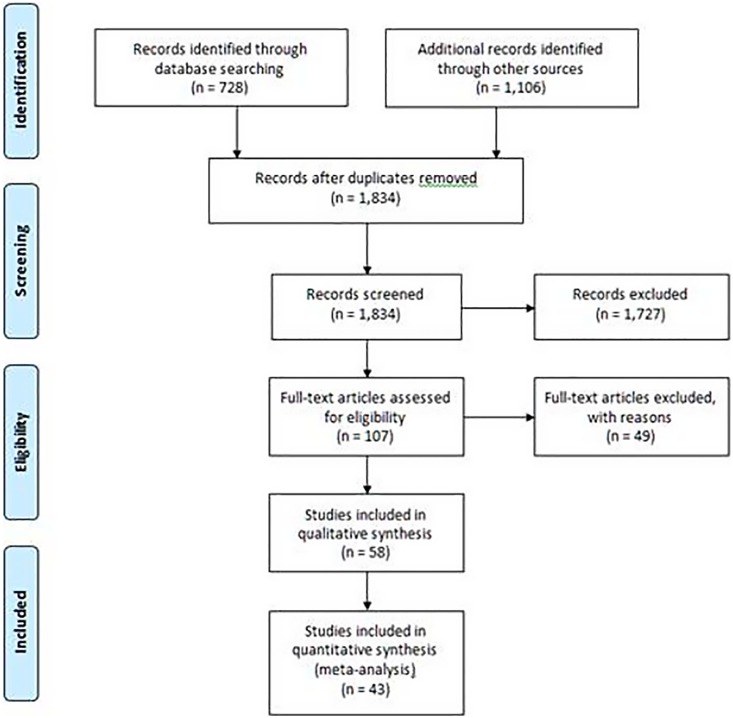
PRISMA Flow Diagram.

Figure 1 displays the overall summary of the evidence search; we could not retrieve three studies for full article review [21]–[23]. Our searches identified all 24 studies included in the systematic review published in Spanish [7]. We included 58 studies that examined 55,766 patients [24]–[81]. One study [28] utilized two designs, hence 59 studies are mentioned in the rest of the manuscript. The overall prevalence of GAS infection was 28.2% (15,254/54,098 patients) (range 3.7% to 66.6%); we did not include one study [28] in the prevalence calculation as only patients with positive cultures were reported (n = 1,688).

### Characteristics of Included Studies

The Table S1 in File S1 displays the overall study characteristics. The study design was prospective in all but eight (11.9%) studies [39], [40], [50]–[52], [55], [63], [66], most were in the pediatric population (n = 35, 59.3%). The setting was solely in outpatient areas (n = 37, 62.7%) or emergency room settings (n = 19, 32.2%). Point of care testing was done in 27 studies (45.6%). Commercial funding was acknowledged in 16 studies (27.2%) [33], [36], [41], [48], [49], [57]–[61], [64], [68], [70], [71], [78], [80]. The Table S2 in File S1 displays the main study characteristics for each study.

### Quality of Included Studies (Risk of Bias)

**Figure 2:**
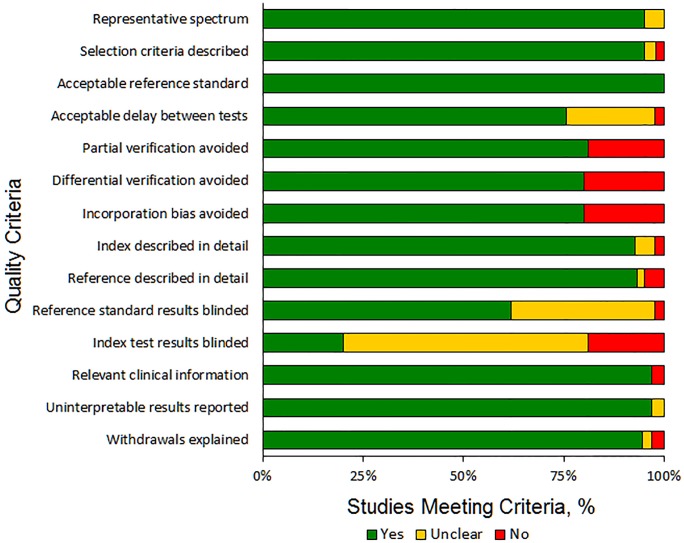
Quality Assessment of Diagnostic Accuracy Studies (QUADAS) assessments of the quality of included studies.

The overall quality of the studies using the QUADAS criteria is shown in Figure 2; in 48 (81.4%) studies partial verification bias was avoided, in 47 (79.7%) studies differential verification bias was avoided, and in 47 (79.7%) studies incorporation bias was avoided (Figure 2). The quality assessment for each study is shown in the Table S3 in File S1. The funnel plot shown in Figure S1 in File S2 was asymmetric and the regression line was not vertical, suggesting the presence of publication bias (p<0.001). In the absence of publication bias, studies of smaller sample size would have a wider distribution of results due to random variation as compared to studies with larger sample size.

### Analyses – Quantitative, Qualitative, and Heterogeneity

The operating test characteristics of the studies are shown in the Table S4 in File S1. The sensitivity ranged from 44% to 100%. The specificity ranged from 69% to 100%.

We explored heterogeneity a-priori by examining studies of highest quality (those with at least 50 patients, prospective data collection, and no verification or differential verification or incorporation biases). Of the 59 studies, 43 (72.9%; 18,464 patients) fulfilled the higher quality definition [24]–[29], [31]–[34], [36]–[38], [41]–[46], [48], [49], [53], [54], [56], [57], [59]–[62], [64], [65], [67]–[71], [73], [75]–[79], [81] and 16 (27.1%; 35,634 patients) did not [28], [30], [35], [39], [40], [47], [50], [51], [58], [63], [66], [72], [74], [80]. The coupled forest plots for sensitivity and specificity and HSROC are shown for higher quality studies (Figure S2 in File S2, Figure S4 in File S2) and lower quality studies (Figure S3 in File S2, Figure S4 in File S2). Both, higher and lower quality studies were highly heterogeneous as demonstrated by high inconsistency values and confidence intervals in the forest plots and wide prediction regions in the HSROC. Also, the summary ROCs have a shoulder-like appearance, suggesting a threshold effect for both higher and lower quality studies.

### Exploratory Analyses- Higher Quality Studies – Pediatrics and Adults Strata

Among the higher quality studies, immunochromatographic methods were described in 34 strata (28 pediatric, six adults), in five enzyme immunoassay strata (three pediatric, two adults), and in four optical immunoassay methods (three pediatric, one adult). The summary of diagnostic accuracy estimates for studies of higher methodological quality is shown in Table 1.

**Table 1:** Summary of diagnostic accuracy estimates, higher study methodological quality*.

Type of test	Pediatrics	Adults
Immunochromatographic		
Number of patients	10,325	1,216
Number of strata	28	6
Sensitivity, %	86 (85–87)	91 (87–94)
Specificity, %	96 (95–96)	93 (92–95)
Inconsistency (*I^2^*)		
- Sensitivity	88%	61%
- Specificity	86%	72%
Enzyme Immunoassay (EIA)		
Number of patients	342	333
Number of strata	3	2
Sensitivity, %	86 (79–92)	86 (81–91)
Specificity, %	92 (88–95)	97 (96–99)
Inconsistency (*I^2^*)		
- Sensitivity	0%	88%
- Specificity	55%	88%
Optical immunoassay (OIA)		
Number of patients	3,294	81
Number of strata	3	1
Sensitivity, %	80 (77–82)	94 (80–99)
Specificity, %	93 (92–94)	69 (54–81)
Inconsistency (*I^2^*)		
- Sensitivity	67%	-
- Specificity	90%	-

*Numbers in parenthesis are 95% confidence intervals.

### Pediatrics -Immunochromatographic Methods

The prevalence of GAS infection in the 28 pediatrics strata was 29.7% (3,062/10,325 patients) (range 11.0% to 66.6%). The studies were of high methodological quality, four studies met all 14 criteria [36], [48], [49], [78], nine met 13 criteria [26], [31], [34], [37], [38], [41], [43], [46], [65], three met 12 criteria [25], [32], [71], and two met 10 criteria [24], [57] (Table S3 in File S1).

**Figure 3:**
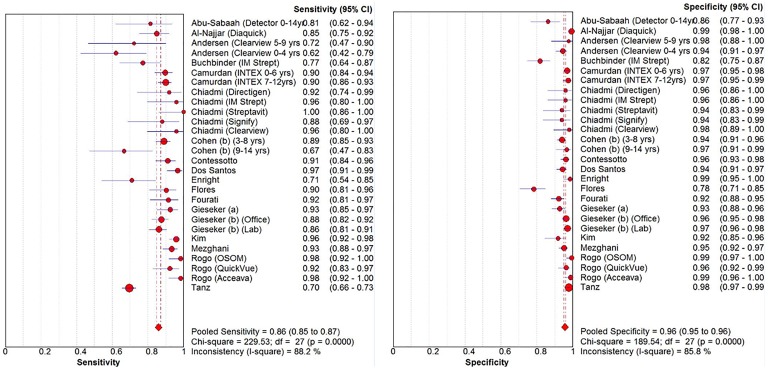
Pediatric strata, forest plots for immunochromatographic methods, higher study methodological quality.

The coupled forest plots for sensitivity and specificity shows high heterogeneity (*I^2^* = 88% for sensitivity and *I^2^* = 86% for specificity; Figure 3, Table 1). As mentioned in the Methods section, we explored additional variables that may explain heterogeneity. In three of the 28 strata, the testing was performed in the laboratory. None of the 28 strata reported Centor or McIssac score. Supplementary figures show HSROC subgroups, no single variable yielded homogenous groups, sponsorship (Figure S5 in File S2), location of care (Figure S6 in File S2), publication year (Figure S7 in File S2), prevalence (Figure S8 in File S2), and region (Figure S9 in File S2). The sensitivity and specificity of the studies remained heterogeneous (large prediction regions) regardless of sponsorship, studies conducted in emergency rooms, studies published more contemporarily, studies with higher GAS infection prevalence, and studies conducted in North America and Europe. Meta-regression showed that in the univariate analyses all strata mentioned above but prevalence of GAS infection by tertile were significant predictors for heterogeneity for both sensitivity and specificity. However, in the joint model, outpatient setting (p = 0.03) and prevalence of GAS infection by tertile prevalence of GAS (p = 0.05) were the only significant variables (data not shown).

Among the 28 high quality studies in the pediatrics strata and immunochromatographic methods, the sensitivity was over 90% in 14 strata (n = 3,362 patients; prevalence of GAS infection, median 32% [Q1–Q3, 28–33%]) [34], [37], [38], [43], [46], [48], [57], [65], [71], between 80–90% in eight strata (n = 4,277 patients; prevalence of GAS infection, median 25% [Q1–Q3, 24–36%]) [24], [25], [32], [34], [36], [49], and less than 80% in six strata (n = 2,685 patients; prevalence of GAS infection, median 25% [Q1–Q3, 21–29%]) [26], [31], [36], [41], [78].

Among the 28 high quality studies in the pediatrics strata and immunochromatographic methods, the specificity was over 95% in 17 strata (n = 7,451 patients; prevalence of GAS infection, median 29% [Q1–Q3, 25–33%]) [25], [26], [32], [34], [36], [37], [41], [49], [65], [71], [78], >90–95% in eight strata (n = 2,340 patients; prevalence of GAS infection, median 32% [Q1–Q3, 22–36%]) [26], [34], [36], [38], [46], [48], [57], 80–90% in two strata (n = 323 patients; prevalence of GAS, 25–26%) [24], [31], and less than 80% in one strata (n = 211 patients; prevalence of GAS infection, 34%) [43].

### Pediatrics - Enzyme Immunoassay and Optical Immunoassay Methods

**Figure 4:**
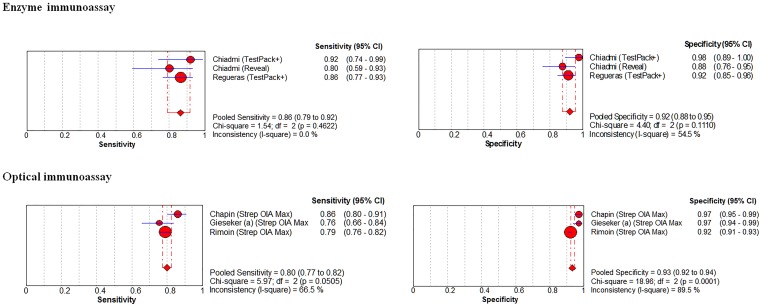
Pediatric strata, forest plots for enzyme immunoassay (EIA, top panels) and optical immunoassay (OIA, bottom panels) methods to diagnose group A streptococcal pharyngitis, higher study methodological quality.

The prevalence of GAS infection in the 3 enzyme immunoassay pediatric strata was 36.3% (124/342 patients) (range 33.3% to 38.5%). The coupled forest plots for sensitivity and specificity shows no or little heterogeneity (*I^2^* = 0% for sensitivity and *I^2^* = 55% for specificity; Figure 4, top panels; Table 1). The pooled sensitivity was 86% (95% CI, 79–92%) and the pooled specificity was 92% (95% CI, 88–95%).

The prevalence of GAS infection in the 3 optical immunoassay pediatric strata was 29.7% (977/3,294 patients) (range 28.7% to 33.3%). The coupled forest plots for sensitivity and specificity shows moderate to high heterogeneity (*I^2^* = 67% for sensitivity and *I^2^* = 90% for specificity; Figure 4; bottom panels, Table 1). The pooled sensitivity was 80% (95% CI, 77–82%) and the pooled specificity was 93% (95% CI, 92–94%).

### Adults - Immunochromatographic Methods

**Figure 5:**
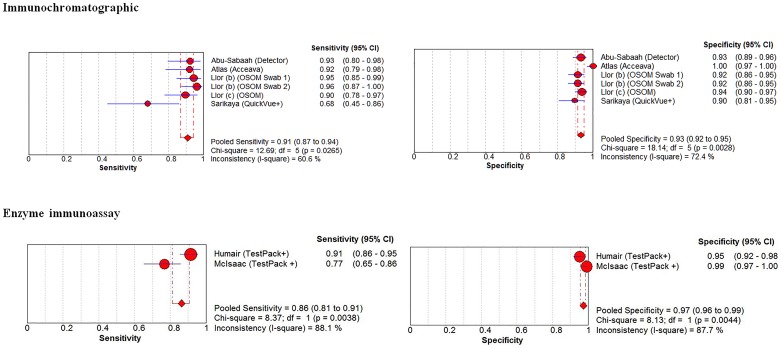
Adult strata, forest plots for immunochromatographic (top panels), enzyme immunoassay (EIA, middle panels) and optical immunoassay (OIA, bottom panels) methods to diagnose group A streptococcal pharyngitis, higher study methodological quality.

The prevalence of GAS infection in the 6 adults strata was 21.3% (259/1,216 patients) (range 16.1% to 25.7%). The coupled forest plots for sensitivity and specificity shows modest heterogeneity (*I^2^* = 61% for sensitivity and *I^2^* = 72% for specificity; Figure 5; top panels, Table 1). The pooled sensitivity was 91% (95% CI, 87–94%) and the pooled specificity was 93% (95% CI, 92 to 95%). One outlier study [75] met 12 quality criteria (Table S3 in File S1) and enrolled 100 patients presenting to an emergency room in Istanbul [75]. Another outlier study, [29] met nine quality criteria (Table S3 in File S1) and enrolled 148 patients presenting to two primary care settings in Boston (Massachusetts).

### Adults - Enzyme immunoassay and Optical Immunoassay Methods

The prevalence of GAS infection in the 2 EIA adult strata was 21.9% (73/333 patients). The coupled forest plots for sensitivity and specificity shows high heterogeneity (*I^2^* = 88% for sensitivity and *I^2^* = 88% for specificity; Figure 5; bottom panels, Table 1). The pooled sensitivity was 86% (95% CI, 81–91%) and the pooled specificity was 97% (95% CI, 96 to 99%).

The prevalence of GAS infection in the single OIA adult strata was 40.7% (33/81 patients), Table 1.

## Discussion

In this systematic review of rapid antigen strep testing, the number of patients included in studies that met high methodological quality criteria was significantly smaller than the number of patients included in lower quality studies (18,464 vs. 35,634, respectively). We also observed publication bias. We could not identify important sources of the high heterogeneity of sensitivity and specificity estimates among higher quality studies using immunochromatographic methods in children (10,325 patients). For higher quality studies using enzyme immunoassay in children (342 patients), the pooled sensitivity was 86% and the pooled specificity was 92% (studies had no or little heterogeneity). In children, immunochromatographic and enzyme immunoassay methods outperform optical immunoassay methods. For the higher quality immunochromatographic methods in the adult population (1,216 patients), the pooled sensitivity was 91% and the pooled specificity was 93%; however, heterogeneity was modest for sensitivity and specificity.

The appropriate diagnosis and management of pharyngitis patients continues to provoke controversy. The controversy exists not just between “experts” but also between guideline panels. Matthys and colleagues reviewed 10 guidelines from both North America and Europe [82]. These guidelines took three different approaches to pharyngitis patients. Some European countries consider pharyngitis a self-limited problem with only rare complications. They eschew testing or antibiotic treatment.

Some guidelines recommend either rapid antigen strep testing or empiric treatment of patients more likely to have GAS pharyngitis, with neither testing nor treatment for patients very unlikely to have GAS infection [82]. Other guidelines aim to limit antibiotic use, and “require” a positive rapid antigen strep test prior to prescribing antibiotics [2].

The debate between the first strategy and the other two strategies rests on a disagreement over the benefits of treating GAS pharyngitis. The debate between the remaining two strategies depends on our estimates of the sensitivity of rapid antigen strep testing and the implications of not treating patients with false negative rapid strep tests.

The profound heterogeneity of the test characteristics among the most studied method, immunochromatographic, represents the major finding of our analysis. When an analysis reveals this degree of heterogeneity then one cannot reliably assign a point estimate to either sensitivity or specificity. Although the pooled estimate of sensitivity (85%; 95% CI 84 to 87%) reported in the systematic review published in Spanish [7] is remarkably similar to the one provided for immunochromatographic methods shown in Table 1 of our study, none can be used as a reliable point estimate given the large heterogeneity observed. While we observed no or little heterogeneity of enzyme immunoassay methods in children, we caution the reader given the relatively small sample size in this group.

Why do these studies show such great heterogeneity for most of the groups? We can only speculate that several factors influence this finding. First, we have a mixture of practical studies in routine clinical settings and research studies with specially trained study personnel. Second, the tests have significant technical variances, as they use different methods to determine the presence of the group A antigen; hence, we could not examine a threshold effect. Finally, evidence suggests the high variability in sensitivity based on clinical spectrum, inoculum size, technical training, and personnel conducting the tests [83]–[85]; the nature of reporting of the studies reviewed precluded further exploration. In our pre-specified approach, we were not able to identify the source of such heterogeneity. Standardization of tests across manufacturers would better define the sensitivity of the RAST. Our finding of great heterogeneity means that we do not have strong confidence in the estimate of sensitivity.

We do not expect this analysis to resolve the ongoing debates about relying on rapid antigen strep testing to make treatment decisions. However, physician decision makers would appreciate accurate estimates of the test characteristics of any test that we use. This study provides estimates of the test characteristics; however, the consistency of performance leaves a broad range of confidence.

Our study has limitations. We could not retrieve three studies for full article review [21]–[23] and we observed publication bias.

## Conclusion

In conclusion, RAST immunochromatographic methods appear to be very sensitive and highly specific to diagnose group A streptococcal pharyngitis among adults but not in children. Using the best evidence, we could not identify important sources of variability of sensitivity and specificity. The present systematic review provides the best evidence for the wide range of sensitivity included in current guidelines.

## Supporting Information

File S1 Contains the following files: **Table S1.** Study Characteristics, Overall (n = 59). **Table S2.** Study Characteristics. **Table S3.** Quality assessment using QUADAS criteria. **Table S4.** Operating Test Characteristics.
(DOCX)

File S2 Contains the following files: **Figure S1.** Funnel plot for rapid antigen tests diagnostic odds ratio. EES = Effective Sample Size. The non-vertical regression line suggests publication bias. The non-vertical regression line suggests publication bias (the results of the studies do not fall into the “funnel” depicted in blue). In the absence of publication bias, studies of smaller sample size would have a wider distribution of Diagnostic Odds Ratios; represented as a wider distribution at the base, which is absent from the plot. **Figure S2.** Forest plots sensitivities and specificities from test accuracy studies of rapid antigen tests to diagnose group A streptococcal pharyngitis for higher study methodological quality. Study test characteristics are sensitivity (left panel) and specificity (right panel). Circles represent the sensitivity or specificity and are proportional to study sample size. Blue lines represent 95% confidence intervals. Diamonds represent pooled estimates of sensitivity or specificity, red lines correspond to their respective 95% confidence intervals. **Figure S3.** Forest plots sensitivities and specificities from test accuracy studies of rapid antigen tests to diagnose group A streptococcal pharyngitis for lower study methodological quality. Study test characteristics are sensitivity (left panel) and specificity (right panel). Circles represent the sensitivity or specificity and are proportional to study sample size. Blue lines represent 95% confidence intervals. Diamonds represent pooled estimates of sensitivity or specificity, red lines correspond to their respective 95% confidence intervals. **Figure S4.** Hierarchical summary receiver-operating characteristic curve plots of rapid antigen tests to diagnose group A streptococcal pharyngitis by study methodological quality. **Figure S5.** Pediatric strata, immunochromatographic methods, higher quality studies. HSROC by sponsorship. **Figure S6.** Pediatric strata, immunochromatographic methods, higher quality studies. HSROC by location of care. **Figure S7.** Pediatric strata, immunochromatographic methods, higher quality studies. HSROC by publication year. **Figure S8.** Pediatric strata, immunochromatographic methods, higher quality studies. HSROC by prevalence. **Figure S9.** Pediatric strata, immunochromatographic methods, higher quality studies. HSROC by region.
(DOCX)

Checklist S1 PRISMA checklist.
(DOC)

Methods S1 (DOCX)
