# Supplementary material for: Rapid Antigen Group A Streptococcus Test to Diagnose Pharyngitis: A Systematic Review and Meta-Analysis
Source: PLoS One. 2014 Nov 4;9(11):e111727. doi: 10.1371/journal.pone.0111727 (PMC4219770; doi:10.1371/journal.pone.0111727)
Supplement: File S1 — Contains the following files: Table S1. Study Characteristics, Overall (n = 59). Table S2. Study Characteristics. Table S3. Quality assessment using QUADAS criteria. Table S4. Operating Test Characteristics. (DOCX) [file pone.0111727.s001.docx]

**Tables Supplementary Information**

**Table S1. Study Characteristics, Overall (n=59)**

**Table S2. Study Characteristics**

**Table S3. Quality assessment using QUADAS criteria**

**Table S4. Operating Test Characteristics**

**Table S1. Study Characteristics, Overall (n=59)**

| **Characteristic*** | **Studies, n (%)** |
| --- | --- |
| Study design  Prospective  Retrospective  Not reported | 51 (86.4%)  7 (11.9%)  1 (1.7%) |
| Population  Pediatrics  Adults  Both  Not reported | 35 (59.3%)  4 (6.8%)  18 (30.5%)  2 (3.4%) |
| Setting  Outpatient  Emergency room  Not reported | 37 (62.7%)  19 (32.2%)  3 (5.1%) |
| Country  USA/ Canada  Europe  Other | 24 (46.7%)  18 (30.5%)  17 (28.8%) |
| Index test location  Point of care  Laboratory  Not reported | 27 (45.6%)  15 (25.4%)  17 (28.8%) |
| Sponsorship, commercial  Yes  No/ not reported | 16 (27.1%)  43 (72.8%) |
| Reference standard, culture  Double swab  Single swab  Not reported | 10 (17.0%)  38 (64.4%)  11 (18.6%) |
| Reference standard, culture media  BAP  BAP / THB  Other  Not reported | 42 (71.2%)  4 (6.8%)  5 (8.5%)  8 (13.6%) |

* Sponsorship, commercial: author employed by funding company, grant, supplies. Other countries: Brazil, Croatia, Latvia, Egypt, Denmark, Finland, France, Greece, Hong Kong, Korea, Norway, Saudi Arabia, Scotland, Spain, Sweden, Switzerland, Tunisia, Turkey, United Arab Emirates. Reference standard, culture media: BAP = THB =. Other culture: BAP + THB + DNA Probe; COBA, SSA Agar, Lim broth, 2nd culture; culture and moderately complex ADT.

**Table S2. Study Characteristics**

| Author (year) | Setting | Age group | Inclusion criteria | Exclusion criteria | Index Test |
| --- | --- | --- | --- | --- | --- |
| Abu-Sabaah  (2006) [^24^](#_ENREF_24) | Emergency room | All | Sore throat. Ages 4-14, >=15yrs. | NR | Detector Strep A Direct Kit |
| Al-Najjar  (2008) | Outpatient | Children | Fever, acute catarrh, acutely inflamed throat/tonsils with or without exudates. | Viral infections, herpetic stomatitis, adenoviral rhino-conjunctivitis, antibiotics within 7 days, cultures for Staph and S. pneumoniae | Diaquick Strep. A Test |
| Andersen  (2003) [^25^](#_ENREF_25) | NR | Children | Tonsillitis. Ages 0-14 yrs. | Antibiotics within 48hrs. | Clearview Strep A |
| Araujo  (2005) [^27^](#_ENREF_27) | Emergency room | Adults | Sore throat, odynophagia, fever, erythema, uvula edema, tonsillar exudate, painful anterior neck nodes, leukocytosis > 12,000/cumm | Cough, rhinorrhea, antibiotics within 14 days | Strep A OIA Max Test |
| Armengol (a)  (2004) [^28^](#_ENREF_28) | Outpatient | Children | Pharyngitis | NR | Mainline Confirms Strep A test |
| Armengol (b)  (2004) [^28^](#_ENREF_28) | Outpatient | Children | Pharyngitis | NR | Mainline Confirms Strep A test |
| Atlas  (2005) [^29^](#_ENREF_29) | Outpatient | Adults | Acute pharyngitis. Age > 18 yrs. | >7 days symptoms, antibiotics within past 24hr, immune-compromised or acute pulmonary flare | Acceava Strep A |
| Ayanruoh  (2009) [^30^](#_ENREF_30) | Emergency room | Children | Pharyngitis. Ages 3-18 years. | Antibiotics within 14 days | Sacks RST |
| Buchbinder  (2007) [^31^](#_ENREF_31) | Emergency room | Children | Sore throat, pharyngitis, fever. | Previous antibiotics | IM-Strep A |
| Camurdan  (2008) [^32^](#_ENREF_32) | Outpatient | Children | Signs and symptoms of acute upper respiratory infection. Ages 4 months - 17 years. | NR | INTEX Strep A Test II |
| Chapin  (2002) [^33^](#_ENREF_33) | Outpatient | Children | Pharyngitis | NR | Strep A OIA Max Test |
| Chiadmi  (2004) [^34^](#_ENREF_34) | Outpatient | Children | Pharyngotonsillitis (fever, sore throat, pharyngitis). Ages 8-14 years. | Antibiotics within 7 days | Signify Rapid Strep A |
| Cohen (a)  (2004) [^35^](#_ENREF_35) | Outpatient | Children | Pharyngitis | NR | Rapid Antigen Tests (not specified) |
| Cohen (b)  (2012) [^36^](#_ENREF_36) | Outpatient | Children | Pharyngitis. Ages 3-15yrs. | Antibiotic within last 7 days | StreptATest |
| Contessotto  (2000) [^37^](#_ENREF_37) | Outpatient | Children | Acute pharyngitis/ tonsillitis, consider antibiotic use. Ages 6m-14 yrs. | Antibiotics prior 3-10 days, immunodeficiency, other infection requiring antibiotics (otitis, sinusitis) | Quick Vue Flex Strep A |
| dos Santos  (2005) [^38^](#_ENREF_38) | Emergency room | Children | Painful throat, dysphagia, fever, palpable glands, painful glands, hyperemia, edema, exudate from the palatine tonsils, exanthema scarlatina | Onset symptoms >7 days, rhinorrhea, coryza, conjunctivitis, coughing and/or sneezing | Quickvue+ Strep A |
| DiMatteo  (2001) [^39^](#_ENREF_39) | Emergency room | Adults | Adults receiving RAST | NR | TestPack Plus Strep A |
| Edmonson  (2005) [^40^](#_ENREF_40) | Outpatient | All | All who had RADT: all + RADT, all false negative RADT and random sample who had true-negative. Age <24 years. | No backup culture for negative RADT, no chart could be located | CARDS QS Strep A |
| Enright  (2011) [^41^](#_ENREF_41) | Emergency room | Children | Pharyngitis. Age 0-13 years. | NR | QuickVue In-Line Strep A |
| Ezike  (2005) [^42^](#_ENREF_42) | Emergency room | Children | Pharyngitis with or without fever, exudates, lymph node enlargement. Ages 5-18 years | Rhinorrhea, conjunctivitis, rales, wheezes, difficulty breathing, systemic antibiotics within 14 days | Strep A OIA Max Test |
| Flores  (2010) [^43^](#_ENREF_43) | Emergency room | Children | Sore throat, less than 5 days. Ages 1-14yrs. | Chronic disease, valvular heart disease, tonsillectomy, recurrent, antibiotics past 15 days. | OSOM Strep A Test |
| Fontes  (2007) [^44^](#_ENREF_44) | Emergency room | Children | Pharyngitis. Ages 1-18 years. | Penicillin within 30 days, antibiotics within 15 days | PathoDx Latex Agglut test |
| Forward  (2006) [^45^](#_ENREF_45) | NR | All | Pharyngeal swabs received at laboratory | NR | Strep A Rapid Test Device |
| Fourati  (2009) [^46^](#_ENREF_46) | Outpatient | Children | Sore throat | NR | ALL-Diag |
| Fox  (2006) [^47^](#_ENREF_47) | Emergency room | Children | Sore throat. Ages 3-18 years | Tonsillectomy | Signify Rapid Strep A |
| Gieseker (a)  (2002) [^48^](#_ENREF_48) | Emergency room | Children | Suspicion of streptococcal pharyngitis | NR | OSOM Ultra Strep A Test |
| Gieseker (b)  (2003) [^49^](#_ENREF_49) | Outpatient | Children | Sore throat, cough, runny nose, known streptococcal cohort, antibiotics within 7 days, tonsillar exudates, lower resp infection, otitis media, scarlatina rash, anterior cervical adenopathy | NR | OSOM Ultra Strep A Test |
| Gurol  (2010) [^50^](#_ENREF_50) | Outpatient | All | RAST and culture done during time frame | One or the other done alone, repeated samples | QuickVue Dipstick Strep A |
| Hall  (2004) [^51^](#_ENREF_51) | Emergency room | Children | RADT performed. Ages 3-17 | Treated < 30 days for sore throat | Acceava Strep A |
| Hinfey  (2010) [^52^](#_ENREF_52) | Emergency room | All | Sore throat with a RADT | NR | Binax Now Strep A |
| Humair  (2006) [^53^](#_ENREF_53) | Outpatient | All | Pharyngitis with 2/4 Centor’s criteria. Age > 15 years. | NR | TestPack Plus Strep A |
| Johansson  (2003) [^54^](#_ENREF_54) | Outpatient | All | Sore throat | <4 years, current antibiotic treatment or not completed within 72 hours, complications demanding different management, patients attending clinic on Fridays after last lab delivery | TestPack Plus Strep A |
| Kawakami  (2003) [^55^](#_ENREF_55) | Outpatient | All | Sore throat. Ages 5 months- 87 years. | NR | Quickvue Strep A |
| Keahey  (2002) [^56^](#_ENREF_56) | Emergency room | Children | Sore throat | Antibiotics within 5 days, steroids within 2 months | PathoDx Latex Agglut test |
| Kim  (2009) [^57^](#_ENREF_57) | Outpatient | Children | Suspected bacterial pharyngitis | NR | SD Bioline Strep A RAT |
| Lindbaek  (2004) [^58^](#_ENREF_58) | Outpatient | All | Sore throat less than 7 days | Antibiotics within 14 days | TestPack Plus Strep A |
| Llor (a)  (2008) [^59^](#_ENREF_59) | Outpatient | All | Odynophagia, 2+ Centor criteria | Antibiotics < 2 weeks or no swab | OSOM Strep A Test |
| Llor (b)  (2009) [^60^](#_ENREF_60) | Outpatient | All | >2 Centor’s criteria. Age >14 yrs | Antibiotics within 14 days | OSOM Strep A Test |
| Llor (c)  (2011) [^61^](#_ENREF_61) | Outpatient | All | 1 or more Centor’s criteria. Ages 14-60 years. | Greater than 5 episodes of pharyngitis in 1 year, immunosuppressed, heart valve, rheumatic fever, antibiotics within 15 days, tonsillectomy | OSOM Strep A Test |
| Maltezou  (2008) [^62^](#_ENREF_62) | Outpatient | Children | 1 or more Centor’s criteria. | Antibiotics within 7 days, immunocompromised | Link 2 Strep A Rapid Test |
| Mayes  (2001) [^63^](#_ENREF_63) | Outpatient | Children | Rapid test | Rapid test, not cultured | Qtest |
| McIsaac  (2004) [^64^](#_ENREF_64) | Outpatient | All | Acute sore throat, modified Centor score >2 | NR | TestPack Plus Strep A |
| Mezghani  (2010) [^65^](#_ENREF_65) | Outpatient | Children | Sore throat | Tonsillectomy, antibiotics past 5 days, viral | OSOM Strep A Test |
| Mirza  (2007) [^66^](#_ENREF_66) | Outpatient | Children | NR | NR | Qtest, Signify (combined) |
| Nerbrand  (2002) [^67^](#_ENREF_67) | Outpatient | All | Pharyngotonsillitis on no antibiotics | NR | QuickVue Dipstick Strep A |
| Parviainen  (2011) [^68^](#_ENREF_68) | Outpatient | NR | NR | NR | Test Pack Strep A |
| Regueras  (2012) [^69^](#_ENREF_69) | Outpatient | Children | Sore throat | Recurrent episode, prior antibiotics < 7 days, non-bacterial pharyngitis | TestPack Plus Strep A |
| Rimoin  (2010) [^70^](#_ENREF_70) | Outpatient | Children | Sore throat. Ages 2-12 | Antibiotics within 3 days, history of rheumatic fever or rheumatic heart disease, requiring hospitalization, missing data | Strep A OIA Max Test |
| Rogo  (2011) [^71^](#_ENREF_71) | Outpatient | Children | Pharyngitis | NR | Acceava Strep A |
| Roosevelt  (2001) [^72^](#_ENREF_72) | Emergency room | Children | Sore throat | NR | Signify Rapid Strep A |
| Rosenberg  (2002) [^73^](#_ENREF_73) | Emergency room | All | Ages >3 years old | Prior antibiotics | TestPack Plus Strep A |
| Santos  (2003) [^74^](#_ENREF_74) | Outpatient | Children | Sore throat, fever, lymphadenitis, purulent secretions | NR | TestPack Plus Strep A |
| Sarikaya  (2010) [^75^](#_ENREF_75) | Emergency room | Adults | Pharyngitis | NR | QuickVue+ Strep A test |
| Schmuziger  (2003) [^76^](#_ENREF_76) | NR | All | Acute tonsillopharyngitis. Ages 8-76 yrs. | NR | Strep A OIA Max Test |
| Sheeler  (2002) [^77^](#_ENREF_77) | Outpatient | NR | Streptococcal infection past 28 days. New sore throat, dysphagia, exudates, with or without fever, malaise | NR | TestPack Plus Strep A |
| Tanz  (2009) [^78^](#_ENREF_78) | Outpatient | Children | Sore throat. Age 3-18 years. | NR | QuickVue Dipstick Strep A |
| Uhl  (2003) [^79^](#_ENREF_79) | Outpatient | All | Pharyngitis, uncharacteristic- coryza, hoarse, cough, diarrhea, conjunctivitis, stomatitis, ulcers | NR | Directigen 1-2-3 Group A Strep Test |
| Van Limbergen  (2006) [^80^](#_ENREF_80) | Emergency room | Children | Pharyngitis | NR | QuickVue+ Strep A test |
| Wong  (2002) [^81^](#_ENREF_81) | Emergency room | All | Sore throat | Sore throat after vomiting, prolonged cough, foreign body/corrosive, peritonsillar cellulitis, cold or influenza, antibiotics within 14 days, incomplete data | Accustrip |

NR = Not reported. RADT= Rapid antigen detection test.

**Table S3. Quality assessment using QUADAS criteria**

| Author (year) | Representative spectrum | Selection criteria described | Acceptable reference standard | Acceptable delay between tests | Partial verification avoided | Differential verification avoided | Incorporation bias avoided | Index described in detail | Reference described in detail | Reference standard results blinded | Index test results blinded | Relevant clinical information | Uninterpretable results reported | Withdrawals explained |
| --- | --- | --- | --- | --- | --- | --- | --- | --- | --- | --- | --- | --- | --- | --- |
| Abu-Sabaah  (2006) [^24^](#_ENREF_24) | Yes | Yes | Yes | Unc | Yes | Yes | Yes | Yes | Yes | Unc | Unc | Yes | Unc | Yes |
| Al-Najjar  (2008) | Yes | Yes | Yes | Yes | Yes | Yes | Yes | Yes | Yes | Unc | Unc | Yes | Yes | Yes |
| Andersen  (2003) [^25^](#_ENREF_25) | Yes | Yes | Yes | Yes | Yes | Yes | Yes | Yes | Yes | Yes | Unc | Yes | Yes | Yes |
| Araujo  (2005) [^27^](#_ENREF_27) | Yes | Yes | Yes | Yes | Yes | Yes | Yes | Yes | Yes | Yes | Yes | Yes | Yes | Yes |
| Armengol (a)  (2004) [^28^](#_ENREF_28) | Yes | Yes | Yes | Unc | No | No | No | Yes | Yes | Yes | No | Yes | Yes | Yes |
| Armengol (b)  (2004) [^28^](#_ENREF_28) | Yes | Yes | Yes | Unc | Yes | Yes | Yes | Yes | Yes | Yes | Yes | Yes | Yes | Yes |
| Atlas  (2005) [^29^](#_ENREF_29) | Yes | Yes | Yes | Unc | Yes | Yes | Yes | Unc | No | Unc | Unc | Yes | Yes | Yes |
| Ayanruoh  (2009) [^30^](#_ENREF_30) | Yes | Yes | Yes | Yes | No | No | No | Yes | Yes | Yes | No | Yes | Yes | Yes |
| Buchbinder  (2007) [^31^](#_ENREF_31) | Yes | Yes | Yes | Yes | Yes | Yes | Yes | Yes | Yes | Yes | Unc | Yes | Yes | Yes |
| Camurdan  (2008) [^32^](#_ENREF_32) | Yes | Yes | Yes | Yes | Yes | Yes | Yes | Yes | Yes | Unc | Unc | Yes | Yes | Yes |
| Chapin  (2002) [^33^](#_ENREF_33) | Yes | Yes | Yes | Yes | Yes | Yes | Yes | Yes | Yes | Yes | Unc | Yes | Yes | Yes |
| Chiadmi  (2004) [^34^](#_ENREF_34) | Yes | Yes | Yes | Yes | Yes | Yes | Yes | Yes | Yes | Yes | Unc | Yes | Yes | Yes |
| Cohen (a)  (2004) [^35^](#_ENREF_35) | Yes | Yes | Yes | Yes | No | No | No | Yes | Yes | Yes | No | Yes | Yes | Yes |
| Cohen (b)  (2012) [^36^](#_ENREF_36) | Yes | Yes | Yes | Yes | Yes | Yes | Yes | Yes | Yes | Yes | Yes | Yes | Yes | Yes |
| Contessotto  (2000) [^37^](#_ENREF_37) | Yes | Yes | Yes | Yes | Yes | Yes | Yes | Yes | Yes | Yes | Unc | Yes | Yes | Yes |
| dos Santos  (2005) [^38^](#_ENREF_38) | Yes | Yes | Yes | Yes | No | No | No | Yes | Yes | Yes | No | Yes | Yes | Yes |
| DiMatteo  (2001) [^39^](#_ENREF_39) | Yes | Yes | Yes | Yes | Yes | Yes | Yes | Yes | Yes | Yes | Unc | Yes | Yes | Yes |
| Edmonson  (2005) [^40^](#_ENREF_40) | Yes | Yes | Yes | Yes | No | No | No | Yes | Yes | Yes | No | Yes | Yes | Yes |
| Enright  (2011) [^41^](#_ENREF_41) | Yes | Yes | Yes | Yes | Yes | Yes | Yes | Yes | Yes | Yes | No | Yes | Yes | Yes |
| Ezike  (2005) [^42^](#_ENREF_42) | Yes | Yes | Yes | Yes | Yes | Yes | Yes | Yes | Yes | Yes | Unc | Yes | Yes | Yes |
| Flores  (2010) [^43^](#_ENREF_43) | Yes | Yes | Yes | Yes | Yes | Yes | Yes | Yes | Yes | Yes | Unc | Yes | Yes | Yes |
| Fontes  (2007) [^44^](#_ENREF_44) | Yes | Yes | Yes | Yes | Yes | Yes | Yes | Yes | Yes | Yes | Yes | Yes | Yes | Yes |
| Forward  (2006) [^45^](#_ENREF_45) | Unc | Yes | Yes | Unc | Yes | Yes | Yes | Yes | Yes | Yes | Unc | Yes | Yes | Yes |
| Fourati  (2009) [^46^](#_ENREF_46) | Yes | Yes | Yes | Yes | Yes | Yes | Yes | Yes | Yes | Yes | Unc | Yes | Yes | Yes |
| Fox  (2006) [^47^](#_ENREF_47) | Yes | Yes | Yes | Yes | Yes | Yes | No | Yes | Yes | Unc | Unc | Yes | Yes | Yes |
| Gieseker (a)  (2002) [^48^](#_ENREF_48) | Yes | Yes | Yes | Yes | Yes | Yes | Yes | Yes | Yes | Yes | Yes | Yes | Yes | Yes |
| Gieseker (b)  (2003) [^49^](#_ENREF_49) | Yes | Yes | Yes | Yes | Yes | Yes | Yes | Yes | Yes | Yes | Yes | Yes | Yes | Yes |
| Gurol  (2010) [^50^](#_ENREF_50) | Yes | Yes | Yes | Unc | Yes | Yes | Yes | Yes | Yes | Unc | Unc | Yes | Yes | Yes |
| Hall  (2004) [^51^](#_ENREF_51) | Yes | Yes | Yes | Yes | No | No | No | Yes | Yes | Yes | No | Yes | Yes | Yes |
| Hinfey  (2010) [^52^](#_ENREF_52) | Yes | Yes | Yes | Unc | No | No | No | Unc | Unc | Unc | No | Yes | Yes | Yes |
| Humair  (2006) [^53^](#_ENREF_53) | Yes | Yes | Yes | Yes | Yes | Yes | Yes | Yes | Yes | Yes | Unc | Yes | Yes | Yes |
| Johansson  (2003) [^54^](#_ENREF_54) | Yes | Yes | Yes | Yes | Yes | Yes | Yes | Yes | Yes | Yes | Unc | Yes | Yes | Yes |
| Kawakami  (2003) [^55^](#_ENREF_55) | Yes | No | Yes | Yes | Yes | Yes | Yes | Yes | Yes | Unc | Unc | Yes | Unc | No |
| Keahey  (2002) [^56^](#_ENREF_56) | Yes | Yes | Yes | Yes | Yes | Yes | Yes | Yes | Yes | Yes | Yes | Yes | Yes | Yes |
| Kim  (2009) [^57^](#_ENREF_57) | Yes | Unc | Yes | No | Yes | Yes | Yes | Yes | Yes | Unc | Yes | Unc | Yes | Yes |
| Lindbaek  (2004) [^58^](#_ENREF_58) | Yes | Yes | Yes | Yes | Yes | No | Yes | Yes | Yes | Yes | Yes | Yes | Yes | Yes |
| Llor (a)  (2008) [^59^](#_ENREF_59) | Yes | Yes | Yes | Yes | Yes | Yes | Yes | Yes | Yes | Unc | Unc | Yes | Yes | Yes |
| Llor (b)  (2009) [^60^](#_ENREF_60) | Yes | Yes | Yes | Yes | Yes | Yes | Yes | Yes | Yes | Unc | Unc | Yes | Yes | Yes |
| Llor (c)  (2011) [^61^](#_ENREF_61) | Yes | Yes | Yes | Unc | Yes | Yes | Yes | Yes | Yes | No | No | Yes | Yes | Yes |
| Maltezou  (2008) [^62^](#_ENREF_62) | Yes | Yes | Yes | Yes | Yes | Yes | Yes | Yes | Yes | Yes | Unc | Yes | Yes | No |
| Mayes  (2001) [^63^](#_ENREF_63) | Yes | Unc | Yes | Unc | No | No | No | No | No | Unc | Unc | Yes | Yes | Unc |
| McIsaac  (2004) [^64^](#_ENREF_64) | Yes | Yes | Yes | Unc | Yes | Yes | Yes | Yes | Yes | Unc | Unc | Yes | Yes | Yes |
| Mezghani  (2010) [^65^](#_ENREF_65) | Yes | Yes | Yes | Yes | Yes | Yes | Yes | Yes | Yes | Yes | Unc | Yes | Yes | Yes |
| Mirza  (2007) [^66^](#_ENREF_66) | Yes | Yes | Yes | Yes | No | No | No | Yes | Yes | Yes | No | Yes | Yes | Yes |
| Nerbrand  (2002) [^67^](#_ENREF_67) | Yes | Yes | Yes | Yes | Yes | Yes | Yes | Yes | Yes | Unc | Unc | Yes | Yes | Yes |
| Parviainen  (2011) [^68^](#_ENREF_68) | Unc | Yes | Yes | Unc | Yes | Yes | Yes | Yes | Yes | Yes | Unc | Unc | Yes | Yes |
| Regueras  (2012) [^69^](#_ENREF_69) | Yes | Yes | Yes | Yes | Yes | Yes | Yes | Yes | Yes | Unc | Yes | Yes | Yes | Yes |
| Rimoin  (2010) [^70^](#_ENREF_70) | Yes | Yes | Yes | Yes | Yes | Yes | Yes | Yes | Yes | Yes | Unc | Yes | Yes | Yes |
| Rogo  (2011) [^71^](#_ENREF_71) | Yes | Yes | Yes | Yes | Yes | Yes | Yes | Yes | Yes | Unc | Unc | Yes | Yes | Yes |
| Roosevelt  (2001) [^72^](#_ENREF_72) | Yes | Yes | Yes | Yes | No | No | No | Yes | Yes | Yes | Yes | Yes | Yes | Yes |
| Rosenberg  (2002) [^73^](#_ENREF_73) | Yes | Yes | Yes | Unc | Yes | Yes | Yes | Yes | Yes | Unc | Unc | Yes | Yes | Yes |
| Santos  (2003) [^74^](#_ENREF_74) | Yes | Yes | Yes | Yes | Yes | Yes | Yes | Yes | Yes | Unc | Unc | Yes | Yes | Yes |
| Sarikaya  (2010) [^75^](#_ENREF_75) | Yes | Yes | Yes | Yes | Yes | Yes | Yes | Yes | Yes | Unc | Unc | Yes | Yes | Yes |
| Schmuziger  (2003) [^76^](#_ENREF_76) | Unc | Yes | Yes | Yes | Yes | Yes | Yes | Yes | Yes | Yes | Unc | Yes | Yes | Yes |
| Sheeler  (2002) [^77^](#_ENREF_77) | Yes | Yes | Yes | Unc | Yes | Yes | Yes | Unc | Yes | Unc | Unc | Yes | Yes | Yes |
| Tanz  (2009) [^78^](#_ENREF_78) | Yes | Yes | Yes | Yes | Yes | Yes | Yes | Yes | Yes | Yes | Yes | Yes | Yes | Yes |
| Uhl  (2003) [^79^](#_ENREF_79) | Yes | Yes | Yes | Yes | Yes | Yes | Yes | Yes | Yes | Yes | Unc | Yes | Yes | Yes |
| Van Limbergen  (2006) [^80^](#_ENREF_80) | Yes | Yes | Yes | Yes | No | No | No | Yes | No | Yes | No | Yes | Yes | Yes |
| Wong  (2002) [^81^](#_ENREF_81) | Yes | Yes | Yes | Yes | Yes | Yes | Yes | Yes | Yes | Unc | Unc | Yes | Yes | Yes |

Unc= Unclear

**Table S4. Operating Test Characteristics**

| Author (year) | True  Positive | False  Negative | False  Positive | True  Negative | Sensitivity  (95% confidence interval) | Specificity  (95% confidence interval) | Sample size | Prevalence |
| --- | --- | --- | --- | --- | --- | --- | --- | --- |
| Abu-Sabaah  (2006) [^24^](#_ENREF_24) | 61 | 6 | 23 | 265 | 91.0% (81.5 - 96.6) | 92.0% (88.3-94.9) | 355 | 18.9% |
| Al-Najjar  (2008) | 68 | 12 | 3 | 422 | 85.0% (75.3-92.0) | 99.3% (98.0-99.9) | 505 | 15.8% |
| Andersen  (2003) [^25^](#_ENREF_25) | 36 | 17 | 15 | 285 | 67.9% (53.7-80.1) | 95.0% (91.9-97.2) | 353 | 15.0% |
| Araujo  (2005) [^27^](#_ENREF_27) | 31 | 2 | 15 | 33 | 93.9% (79.8-99.3) | 68.8% (53.7-81.3) | 81 | 40.7% |
| Armengol (a)  (2004) [^28^](#_ENREF_28) | 877 | 111 | - | - | 88.8% (86.6-90.7) | - | 988 | - |
| Armengol (b)  (2004) [^28^](#_ENREF_28) | 581 | 99 | - | - | 85.4% (82.6-88.0) | - | 680 | - |
| Atlas  (2005) [^29^](#_ENREF_29) | 35 | 3 | 0 | 110 | 92.1% (78.6-98.3) | 100% (96.7-100) | 148 | 25.7% |
| Ayanruoh  (2009) [^30^](#_ENREF_30) | 1,474 | 2 | 0 | 5,081 | 99.9% (99.5-100) | 100% (99.9-100) | 6,557 | 22.5% |
| Buchbinder  (2007) [^31^](#_ENREF_31) | 44 | 13 | 29 | 130 | 77.2% (64.2-87.3) | 81.8% (74.9-87.4) | 216 | 26.4% |
| Camurdan  (2008) [^32^](#_ENREF_32) | 426 | 49 | 22 | 751 | 89.7% (86.6-92.3) | 97.2% (95.7-98.2) | 1,248 | 38.1% |
| Chapin  (2002) [^33^](#_ENREF_33) | 149 | 24 | 10 | 337 | 86.1% (80.1-90.9) | 97.1% (94.8-98.6) | 520 | 33.3% |
| Chiadmi  (2004) [^34^](#_ENREF_34) | 24 | 1 | 2 | 48 | 96.0% (79.6-99.9) | 96.0% (86.3-99.5) | 75 | 33.3% |
| Cohen (a)  (2004) [^35^](#_ENREF_35) | 268 | 7 | 0 | 329 | 97.5% (94.8-99.0) | 100% (98.9-100) | 604 | 45.5% |
| Cohen (b)  (2012) [^36^](#_ENREF_36) | 247 | 38 | 27 | 473 | 86.7% (82.2-90.4) | 94.6% (92.2-96.4) | 785 | 36.3% |
| Contessotto  (2000) [^37^](#_ENREF_37) | 103 | 10 | 11 | 277 | 91.2% (84.3-95.7) | 96.2% (93.3-98.1) | 401 | 28.2% |
| dos Santos  (2005) [^38^](#_ENREF_38) | 89 | 3 | 16 | 268 | 96.7% (90.8-99.3) | 94.4% (91.0-96.7) | 376 | 24.5% |
| DiMatteo  (2001) [^39^](#_ENREF_39) | 115 | 22 | 0 | 361 | 83.9% (76.7-89.7) | 100% (99.0-100) | 498 | 27.5% |
| Edmonson  (2005) [^40^](#_ENREF_40) | 384 | 65 | 0 | 735 | 85.5% (81.9-88.6) | 100% (99.5-100) | 1,184 | 37.9% |
| Enright  (2011) [^41^](#_ENREF_41) | 27 | 11 | 2 | 137 | 71.1% (54.1-84.6) | 98.6% (94.9-99.8) | 177 | 21.5% |
| Ezike  (2005) [^42^](#_ENREF_42) | 71 | 4 | 0 | 102 | 94.7% (86.9-98.5) | 100% (96.4-100) | 177 | 42.4% |
| Flores  (2010) [^43^](#_ENREF_43) | 65 | 7 | 30 | 109 | 90.3% (81.0-96.0) | 78.4% (70.6-84.9) | 211 | 34.1% |
| Fontes  (2007) [^44^](#_ENREF_44) | 49 | 5 | 19 | 156 | 90.7% (79.7-96.9) | 89.1% (83.6-93.3) | 229 | 23.6% |
| Forward  (2006) [^45^](#_ENREF_45) | 123 | 48 | 47 | 610 | 71.9% (64.6-78.5) | 92.8% (90.6-94.7) | 828 | 20.7% |
| Fourati  (2009) [^46^](#_ENREF_46) | 54 | 5 | 18 | 215 | 91.5% (81.3-97.2) | 92.3% (88.1-95.4) | 292 | 20.2% |
| Fox  (2006) [^47^](#_ENREF_47) | 25 | 6 | 0 | 22 | 80.6% (62.5-92.5) | 100% (84.6-100) | 53 | 58.5% |
| Gieseker (a)  (2002) [^48^](#_ENREF_48) | 87 | 7 | 15 | 193 | 92.6% (85.3-97.0) | 92.8% (88.4-95.9) | 302 | 31.1% |
| Gieseker (b)  (2003) [^49^](#_ENREF_49) | 184 | 26 | 25 | 642 | 87.6% (82.4-91.8) | 96.3% (94.5-97.6) | 877 | 23.9% |
| Gurol  (2010) [^50^](#_ENREF_50) | 51 | 28 | 12 | 362 | 64.6% (53.0-75.0) | 96.8% (94.5-98.3) | 453 | 17.4% |
| Hall  (2004) [^51^](#_ENREF_51) | 117 | 35 | 0 | 409 | 77.0% (69.5-83.4) | 100% (99.1-100) | 561 | 27.1% |
| Hinfey  (2010) [^52^](#_ENREF_52) | 202 | 261 | 3 | 998 | 43.6% (39.1-48.3) | 99.7% (99.1-99.9) | 1,464 | 31.6% |
| Humair  (2006) [^53^](#_ENREF_53) | 128 | 12 | 11 | 221 | 91.4% (85.5-95.5) | 95.3% (91.7-97.6) | 372 | 37.6% |
| Johansson  (2003) [^54^](#_ENREF_54) | 46 | 7 | 4 | 116 | 86.8% (74.7-94.5) | 96.7% (91.7-99.1) | 173 | 30.6% |
| Kawakami  (2003) [^55^](#_ENREF_55) | 34 | 2 | 0 | 64 | 94.4% (81.3-99.3) | 100% (94.4-100) | 100 | 36% |
| Keahey  (2002) [^56^](#_ENREF_56) | 65 | 10 | 18 | 72 | 86.7% (76.8-93.4) | 80.0% (70.2-87.7) | 165 | 45.5% |
| Kim  (2009) [^57^](#_ENREF_57) | 187 | 8 | 8 | 90 | 95.9% (92.1-98.2) | 91.8% (84.5-96.4) | 293 | 66.6% |
| Lindbaek  (2004) [^58^](#_ENREF_58) | 106 | 4 | 27 | 169 | 96.4% (91.0-99.0) | 86.2% (80.6-90.7) | 306 | 35.9% |
| Llor (a)  (2008) [^59^](#_ENREF_59) | 38 | 2 | 10 | 132 | 95.0% (83.1-99.4) | 93.0% (87.4-96.6) | 182 | 22.0% |
| Llor (b)  (2009) [^60^](#_ENREF_60) | 52 | 3 | 14 | 153 | 94.5% (84.9-98.9) | 91.6% (86.3-95.3) | 222 | 24.8% |
| Llor (c)  (2011) [^61^](#_ENREF_61) | 44 | 5 | 14 | 213 | 89.8% (77.8-96.6) | 93.8% (89.9-96.6) | 276 | 17.8% |
| Maltezou  (2008) [^62^](#_ENREF_62) | 103 | 21 | 22 | 286 | 83.1% (75.3-89.2) | 92.9% (89.4-95.5) | 432 | 28.7% |
| Mayes  (2001) [^63^](#_ENREF_63) | 1,299 | 132 | 0 | 3,342 | 90.8% (89.2-92.2) | 100% (99.9-100) | 4,773 | 30.0% |
| McIsaac  (2004) [^64^](#_ENREF_64) | 189 | 39 | 5 | 554 | 82.9% (77.4-87.5) | 99.1% (97.9-99.7) | 787 | 29.0% |
| Mezghani  (2010) [^65^](#_ENREF_65) | 155 | 11 | 16 | 322 | 93.4% (88.5-96.6) | 95.3% (92.4-97.3) | 504 | 32.9% |
| Mirza  (2007) [^66^](#_ENREF_66) | 4,342 | 968 | 0 | 13,199 | 81.8% (80.7-82.8) | 100% (100-100) | 18,509 | 28.7% |
| Nerbrand  (2002) [^67^](#_ENREF_67) | 61 | 21 | 60 | 394 | 74.4% (63.6-83.4) | 86.8% (83.3-89.8) | 536 | 15.3% |
| Parviainen  (2011) [^68^](#_ENREF_68) | 33 | 5 | 12 | 181 | 86.8% (71.9-95.6) | 93.8% (89.4-96.7) | 231 | 16.5% |
| Regueras  (2012) [^69^](#_ENREF_69) | 64 | 10 | 10 | 108 | 86.5% (76.5-93.3) | 91.5% (85.0-95.9) | 192 | 38.5% |
| Rimoin  (2010) [^70^](#_ENREF_70) | 561 | 149 | 136 | 1,626 | 79.0% (75.8-82.0) | 92.3% (90.9-93.5) | 184 | 24.5% |
| Rogo  (2011) [^71^](#_ENREF_71) | 63 | 1 | 2 | 162 | 98.4% (91.6-100) | 98.8% (95.7-99.9) | 228 | 28.1% |
| Roosevelt  (2001) [^72^](#_ENREF_72) | 76 | 7 | 16 | 223 | 91.6% (83.4-96.5) | 93.3% (89.4-96.1) | 322 | 25.8% |
| Rosenberg  (2002) [^73^](#_ENREF_73) | 24 | 9 | 1 | 93 | 75.0% (56.6-88.5) | 98.9% (94.2-100) | 126 | 25.4% |
| Santos  (2003) [^74^](#_ENREF_74) | 11 | 4 | 2 | 32 | 73.3% (44.9-92.2) | 94.1% (80.3-99.3) | 49 | 30.6% |
| Sarikaya  (2010) [^75^](#_ENREF_75) | 15 | 7 | 8 | 70 | 68.2% (45.1-86.1) | 89.7% (80.8-95.9) | 100 | 22.0% |
| Schmuziger  (2003) [^76^](#_ENREF_76) | 18 | 5 | 0 | 42 | 78.3% (56.3-92.5) | 100% (91.6-100) | 65 | 35.4% |
| Sheeler  (2002) [^77^](#_ENREF_77) | 104 | 10 | 4 | 93 | 91.2% (84.5-95.7) | 95.9% (89.8-98.9) | 211 | 54.0% |
| Tanz  (2009) [^78^](#_ENREF_78) | 385 | 168 | 28 | 1,262 | 69.6% (65.6-73.4) | 97.8% (96.9-98.6) | 1,843 | 30.0% |
| Uhl  (2003) [^79^](#_ENREF_79) | 30 | 25 | 1 | 328 | 54.5% (40.6-68.0) | 99.7% (98.3-100) | 384 | 14.3% |
| Van Limbergen  (2006) [^80^](#_ENREF_80) | 21 | 11 | 1 | 168 | 65.6% (46.8-81.4) | 99.4% (96.7-100) | 201 | 15.9% |
| Wong  (2002) [^81^](#_ENREF_81) | 10 | 9 | 9 | 486 | 52.6% (28.9-75.6) | 98.2% (96.6-99.2) | 514 | 3.7% |
