# Supplementary material for: Rapid Antigen Group A Streptococcus Test to Diagnose Pharyngitis: A Systematic Review and Meta-Analysis
Source: PLoS One. 2014 Nov 4;9(11):e111727. doi: 10.1371/journal.pone.0111727 (PMC4219770; doi:10.1371/journal.pone.0111727)

**Supporting Figures Legends**

**Figure S1. Funnel plot for rapid antigen tests diagnostic odds ratio. EES = Effective Sample Size. The non-vertical regression line suggests publication bias.**

The non-vertical regression line suggests publication bias (the results of the studies do not fall into the “funnel” depicted in blue). In the absence of publication bias, studies of smaller sample size would have a wider distribution of Diagnostic Odds Ratios; represented as a wider distribution at the base, which is absent from the plot.

**Figure S2. Forest plots sensitivities and specificities from test accuracy studies of rapid antigen tests to diagnose group A streptococcal pharyngitis for higher study methodological quality.**

Study test characteristics are sensitivity (left panel) and specificity (right panel). Circles represent the sensitivity or specificity and are proportional to study sample size. Blue lines represent 95% confidence intervals. Diamonds represent pooled estimates of sensitivity or specificity, red lines correspond to their respective 95% confidence intervals.

**Figure S3. Forest plots sensitivities and specificities from test accuracy studies of rapid antigen tests to diagnose group A streptococcal pharyngitis for lower study methodological quality.**

Study test characteristics are sensitivity (left panel) and specificity (right panel). Circles represent the sensitivity or specificity and are proportional to study sample size. Blue lines represent 95% confidence intervals. Diamonds represent pooled estimates of sensitivity or specificity, red lines correspond to their respective 95% confidence intervals.

**Figure S4. Hierarchical summary receiver-operating characteristic curve plots of rapid antigen tests to diagnose group A streptococcal pharyngitis by study methodological quality.**

**Figure S5. Pediatric strata, immunochromatographic methods, higher quality studies. HSROC by sponsorship.**

**Figure S6. Pediatric strata, immunochromatographic methods, higher quality studies. HSROC by location of care.**

**Figure S7. Pediatric strata, immunochromatographic methods, higher quality studies. HSROC by publication year.**

**Figure S8. Pediatric strata, immunochromatographic methods, higher quality studies. HSROC by prevalence.**

**Figure S9. Pediatric strata, immunochromatographic methods, higher quality studies. HSROC by region.**

**Figure S1. Funnel plot for rapid antigen tests diagnostic odds ratio. EES = Effective Sample Size. The non-vertical regression line suggests publication bias.**

The non-vertical regression line suggests publication bias (the results of the studies do not fall into the “funnel” depicted in blue). In the absence of publication bias, studies of smaller sample size would have a wider distribution of Diagnostic Odds Ratios; represented as a wider distribution at the base, which is absent from the plot.

**
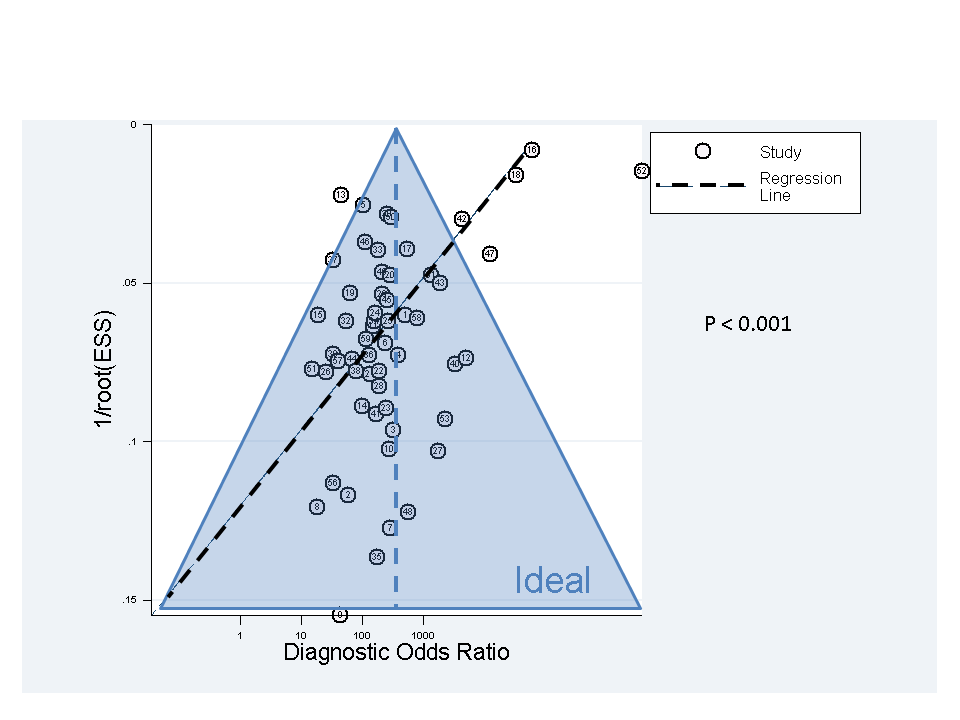
**

**Figure S2. Forest plots sensitivities and specificities from test accuracy studies of rapid antigen tests to diagnose group A streptococcal pharyngitis for higher study methodological quality.** Study test characteristics are sensitivity (left panel) and specificity (right panel). Circles represent the sensitivity or specificity and are proportional to study sample size. Blue lines represent 95% confidence intervals. Diamonds represent pooled estimates of sensitivity or specificity, red lines correspond to their respective 95% confidence intervals.

**Figure S3. Forest plots sensitivities and specificities from test accuracy studies of rapid antigen tests to diagnose group A streptococcal pharyngitis for lower study methodological quality.** Study test characteristics are sensitivity (left panel) and specificity (right panel). Circles represent the sensitivity or specificity and are proportional to study sample size. Blue lines represent 95% confidence intervals. Diamonds represent pooled estimates of sensitivity or specificity, red lines correspond to their respective 95% confidence intervals.

**Figure S4. Hierarchical summary receiver-operating characteristic curve plots of rapid antigen tests to diagnose group A streptococcal pharyngitis by study methodological quality.**

**Higher Quality Studies Lower Quality Studies**

**
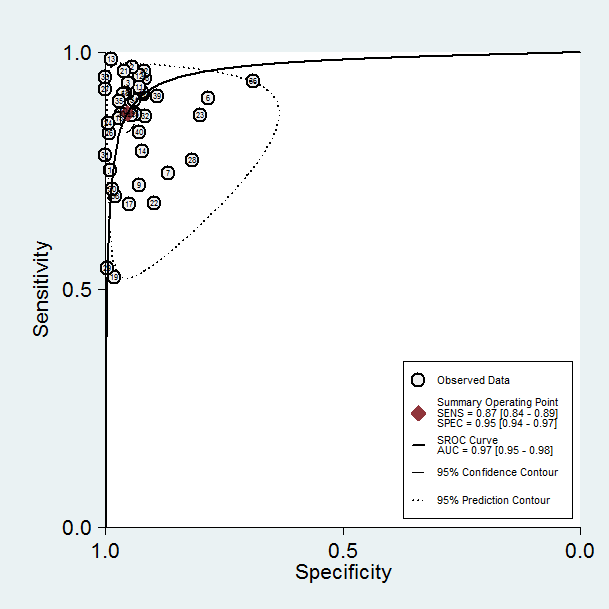

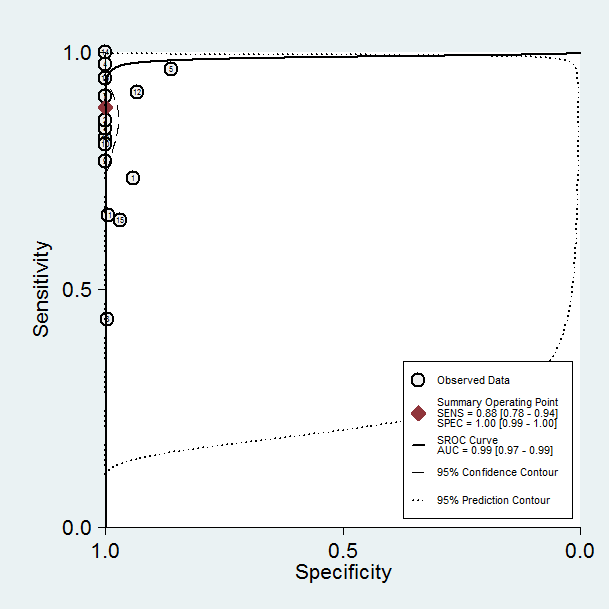
**

**Figure S5. Pediatric strata, immunochromatographic methods, higher quality studies. HSROC by sponsorship.**

**Commercial None**


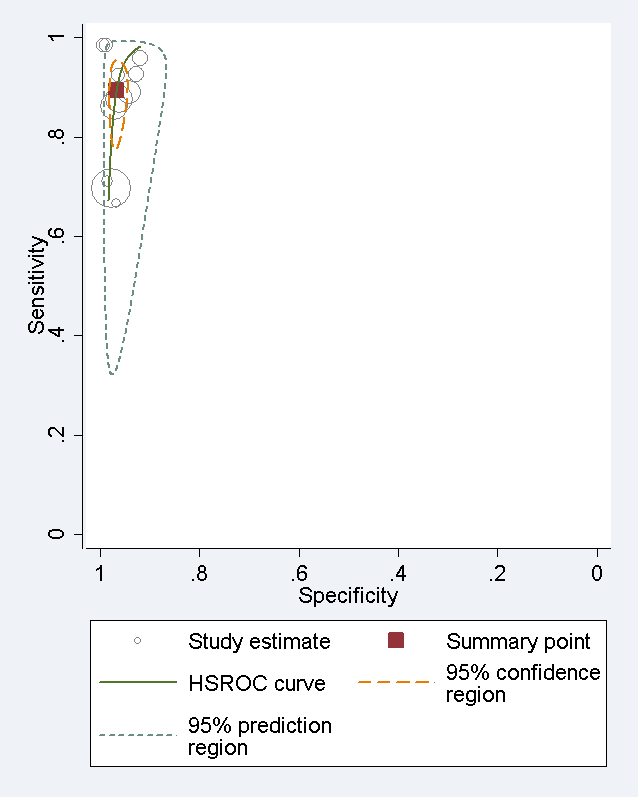

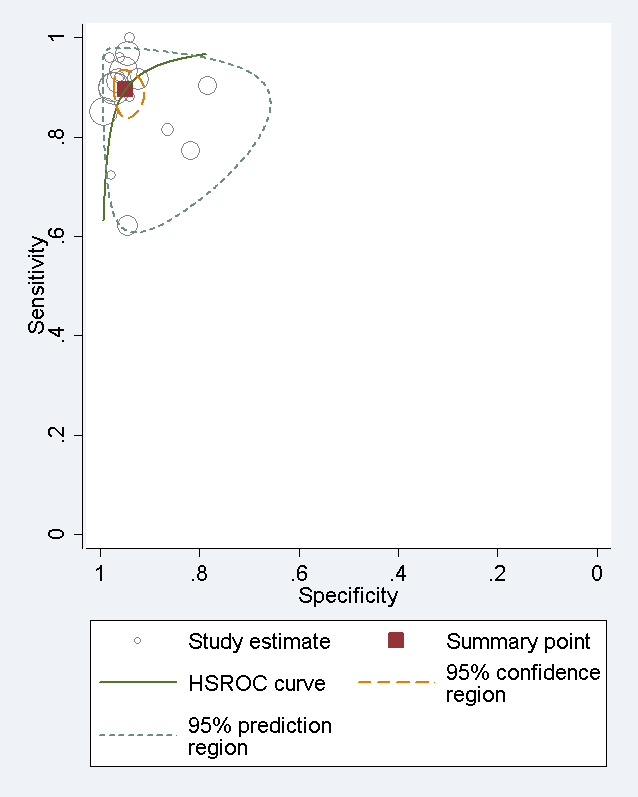


**Figure S6. Pediatric strata, immunochromatographic methods, higher quality studies. HSROC by location of care.**

**Outpatient Emergency Room**


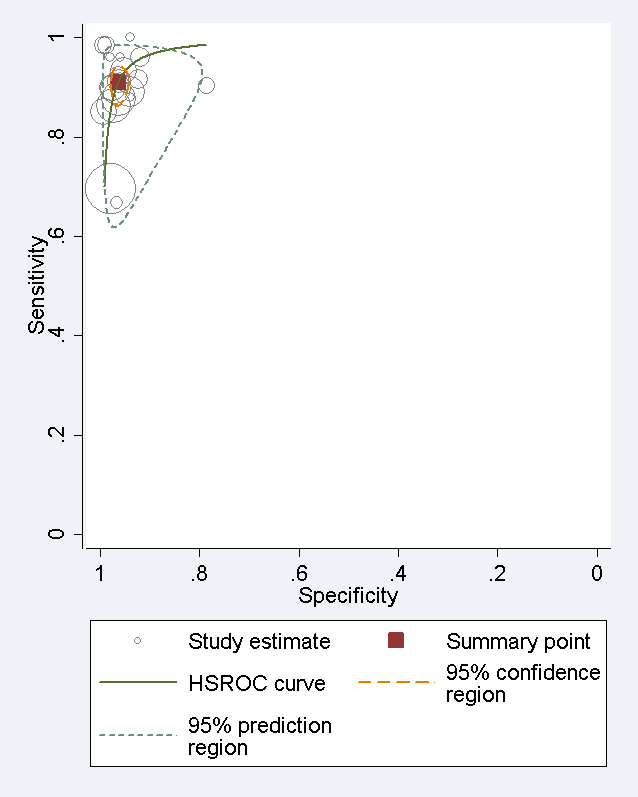

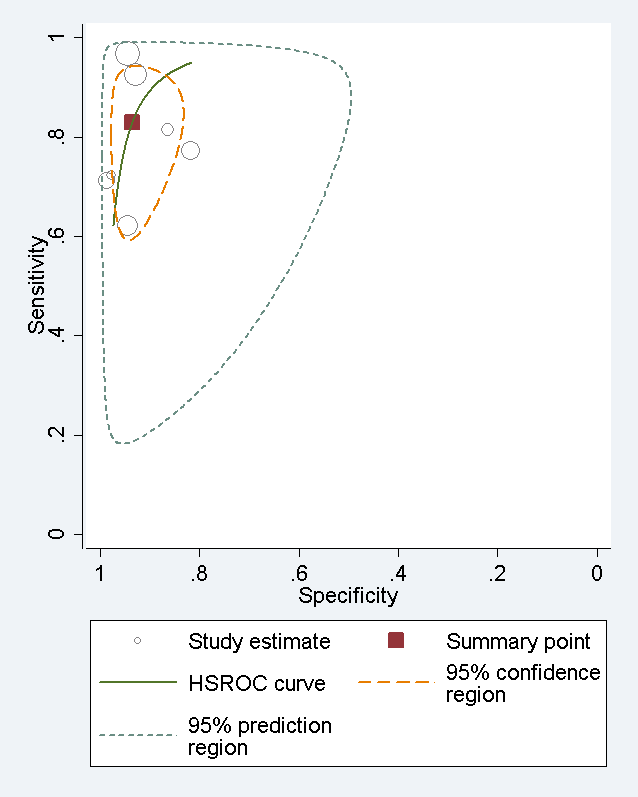


**Figure S7. Pediatric strata, immunochromatographic methods, higher quality studies. HSROC by publication year.**

**2000-05 2006-12**


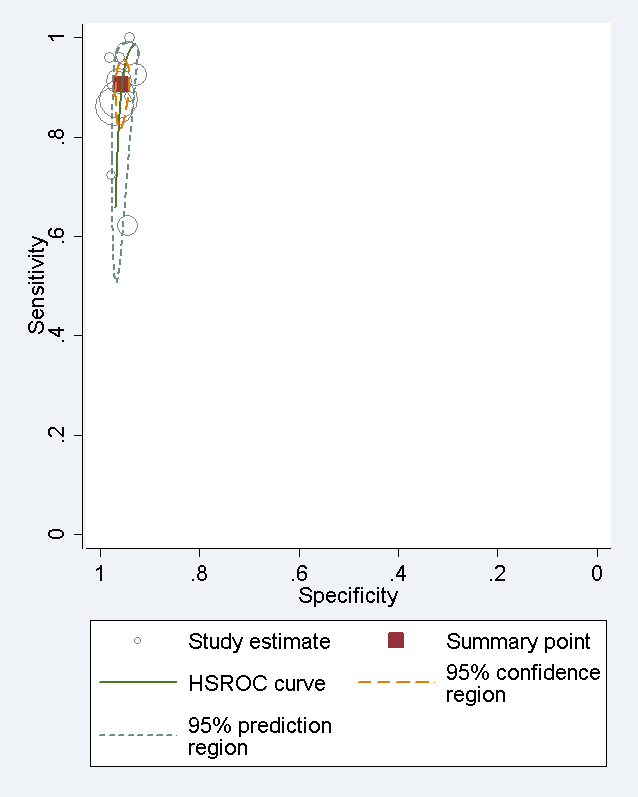

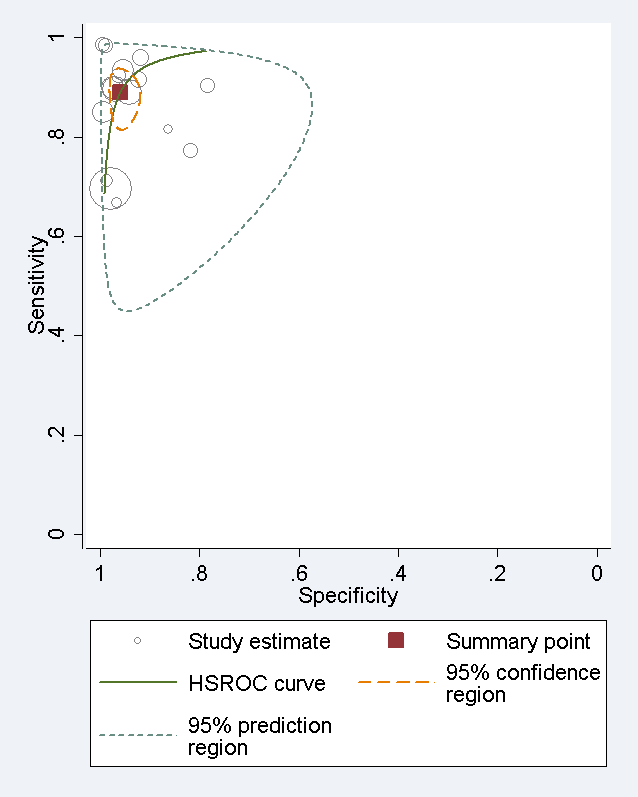


**Figure S8. Pediatric strata, immunochromatographic methods, higher quality studies. HSROC by prevalence.**

**Lowest Tertile Middle Tertile Highest Tertile**


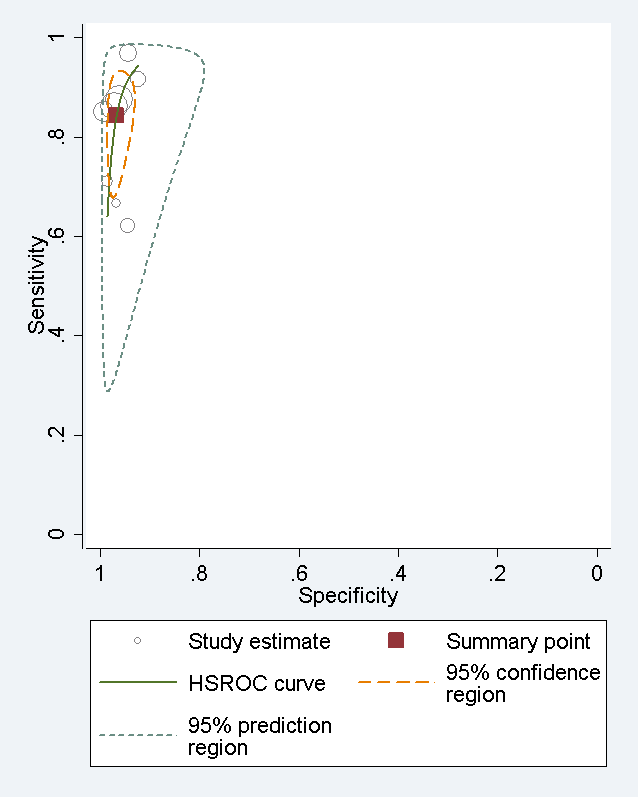

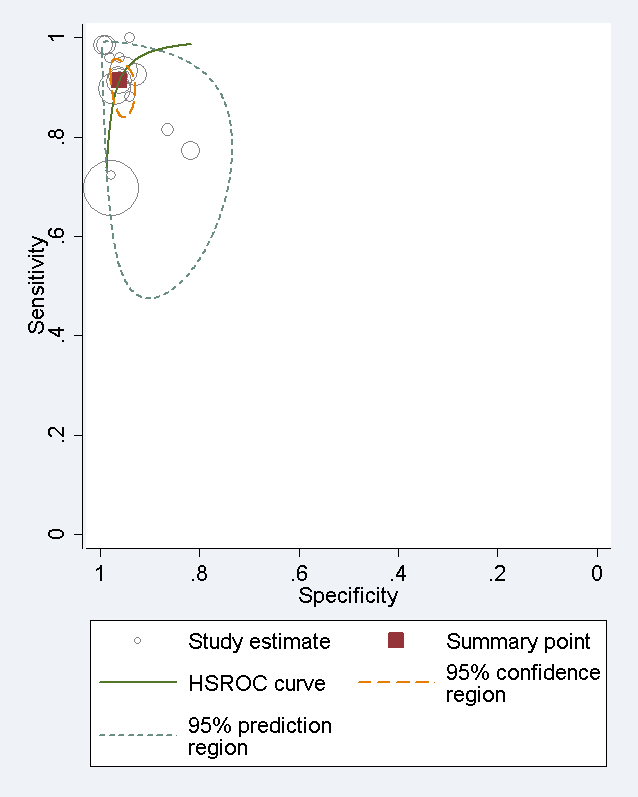

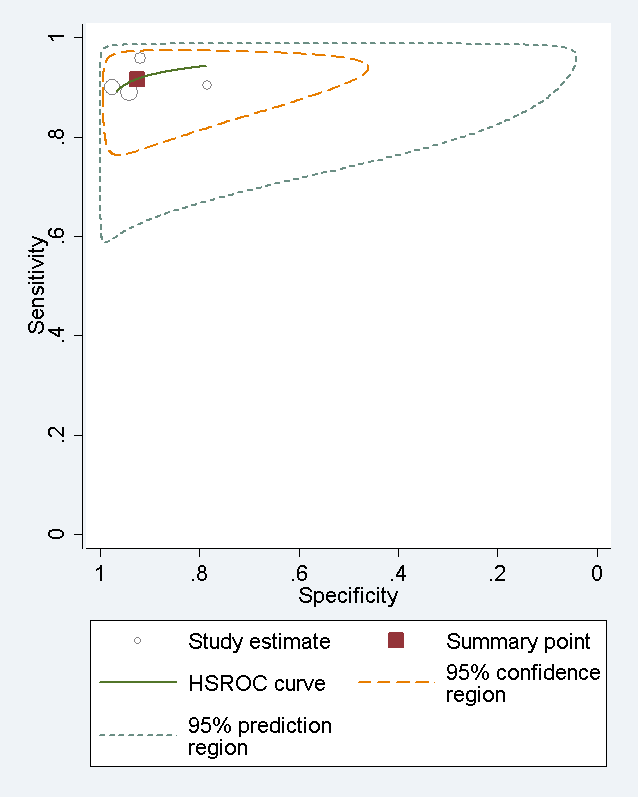


**Figure S9. Pediatric strata, immunochromatographic methods, higher quality studies. HSROC by region.**

**USA – Canada Europe Other**


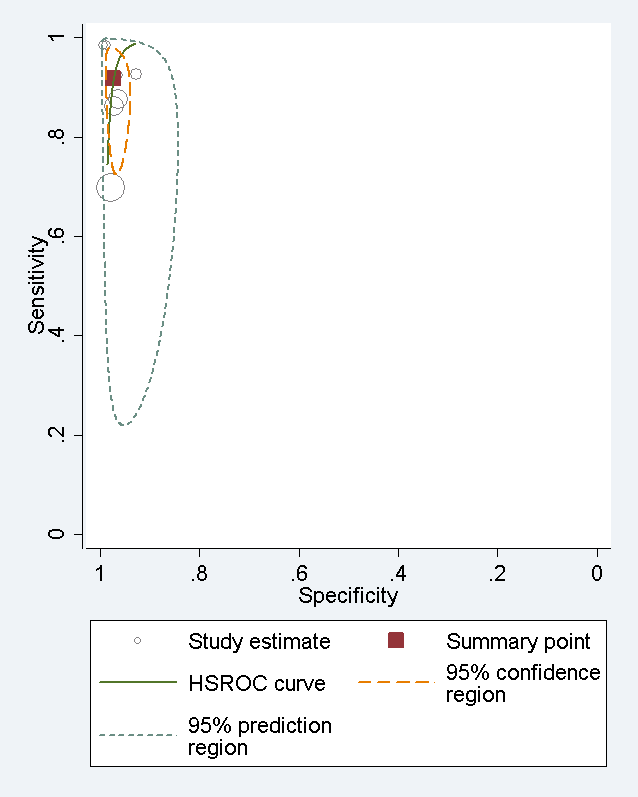

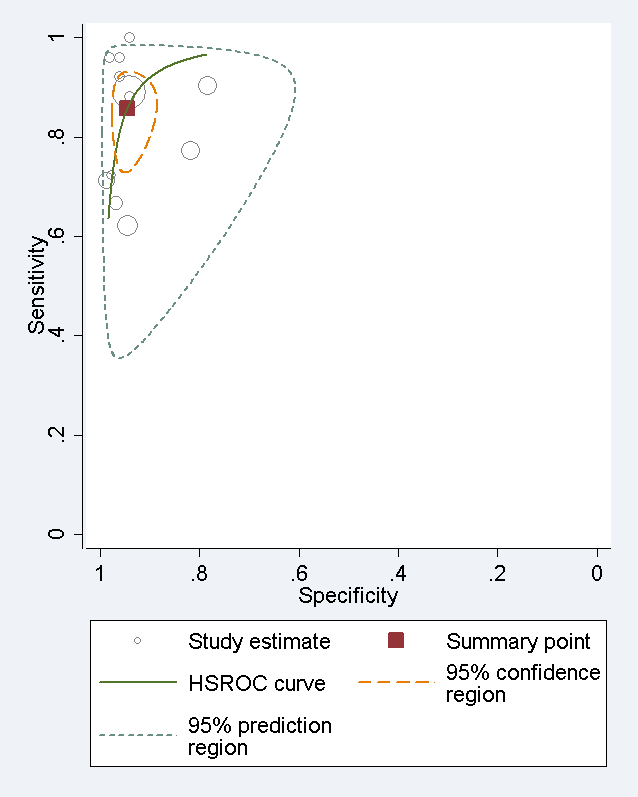

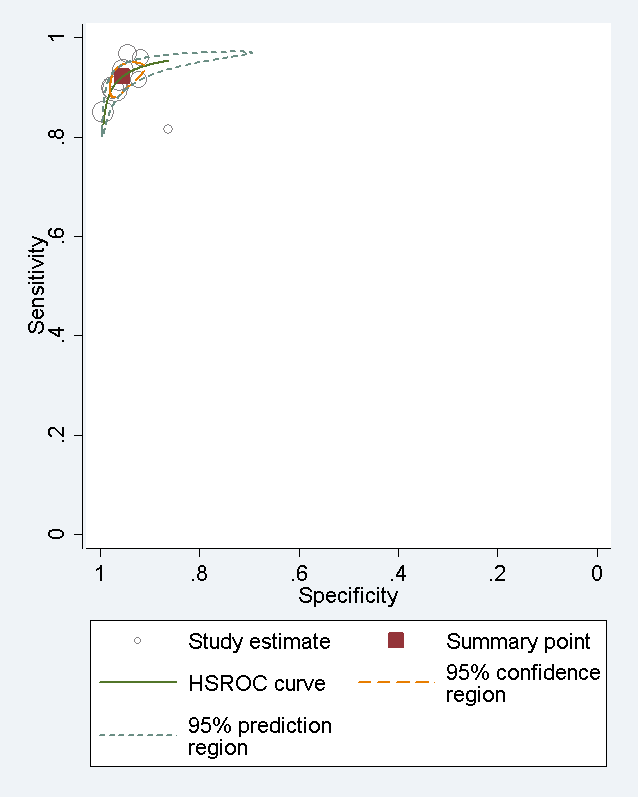

Supplement: File S2 — Contains the following files: Figure S1. Funnel plot for rapid antigen tests diagnostic odds ratio. EES = Effective Sample Size. The non-vertical regression line suggests publication bias. The non-vertical regression line suggests publication bias (the results of the studies do not fall into the “funnel” depicted in blue). In the absence of publication bias, studies of smaller sample size would have a wider distribution of Diagnostic Odds Ratios; represented as a wider distribution at the base, which is absent from the plot. Figure S2. Forest plots sensitivities and specificities from test accuracy studies of rapid antigen tests to diagnose group A streptococcal pharyngitis for higher study methodological quality. Study test characteristics are sensitivity (left panel) and specificity (right panel). Circles represent the sensitivity or specificity and are proportional to study sample size. Blue lines represent 95% confidence intervals. Diamonds represent pooled estimates of sensitivity or specificity, red lines correspond to their respective 95% confidence intervals. Figure S3. Forest plots sensitivities and specificities from test accuracy studies of rapid antigen tests to diagnose group A streptococcal pharyngitis for lower study methodological quality. Study test characteristics are sensitivity (left panel) and specificity (right panel). Circles represent the sensitivity or specificity and are proportional to study sample size. Blue lines represent 95% confidence intervals. Diamonds represent pooled estimates of sensitivity or specificity, red lines correspond to their respective 95% confidence intervals. Figure S4. Hierarchical summary receiver-operating characteristic curve plots of rapid antigen tests to diagnose group A streptococcal pharyngitis by study methodological quality. Figure S5. Pediatric strata, immunochromatographic methods, higher quality studies. HSROC by sponsorship. Figure S6. Pediatric strata, immunochromatographic methods, higher quality stu [file pone.0111727.s002.docx]
