# Supplementary material for: Rapid Antigen Group A Streptococcus Test to Diagnose Pharyngitis: A Systematic Review and Meta-Analysis
Source: PLoS One. 2014 Nov 4;9(11):e111727. doi: 10.1371/journal.pone.0111727 (PMC4219770; doi:10.1371/journal.pone.0111727)
Supplement: Methods S1 — (DOCX) [file pone.0111727.s004.docx]

**Methods Supplementary Information**

*Search Terms*

Target Condition. The medical subject headings (MeSH Terms) combined using the boolean logic operator OR for the target condition concept are: Streptococcal Infections, Streptococcus Pyogenes, or Pharyngitis. The text words used (some with truncation) are: Group A Streptococc*, gabhs , gas, ( Streptococc* AND Pyogenes), Group A Beta-Hemolytic, Group A Beta-Haemolytic, Group A Streptococc*, Group A Beta-Hemolytic Streptococc*, Group A Beta-Haemolytic Streptococc*, The Clinical Queries diagnosis/broad filter using the terms (Streptococc* AND Pyogenes) OR (Steptococc* AND Infection*) OR Pharyngitis was ORed in as well.

Index Test. The medical subject headings (MeSH Terms) combined using the boolean logic operator OR for the index test are: Reagent Kits, Diagnostic, Diagnostic Tests, Routine, Bacteriological Techniques, Immunoassay, or Latex Fixation Tests. Text words (some used with truncation) are: radt, rapid antigen detection test*, rast , rsat, gas antigen test*, negative rapid antigen test*, positive rapid antigen test*, rapid antigen test*, Immunoassay , Biostar Strep A, Strep A Testpack, Quickvue Dipstick, Testpack Strep A, Tandem Icon Strep A, Wellcome Diagnostics Reveal, Strep A Oia, Direct Strep Eia, Quickvue In-Line Strep, Culturette Brand 10 Minute, Card OS Strep, Card OS, Respirastick, Osom Ultra Strep A, Osom Strep A, Visuwell, Directigen 1, 2, 3, Directigen Strep, Test Pack Strep A, Test Pack Plus, or Signify Strep A.

Test Characteristics. The medical subject headings (MeSH Terms) combined using the boolean logic operator OR for the test characteristics terms are: Sensitivity and Specificity, Diagnostic Errors, Likelihood Functions, Reproducibility of Results, Area Under Curve, Probability, or Differential Diagnosis. The text words used (some with truncation) are: False Positive, False Negative*, False Rate*, Likelihood, Receiver Operat*, Observer Variation*, Predictive Value*, or Sensitiv*.
